# Supplementary material for: Oxidative Inactivation of the Proteasome Augments Alveolar Macrophage Secretion of Vesicular SOCS3
Source: Cells. 2020 Jun 30;9(7):1589. doi: 10.3390/cells9071589 (PMC7408579; doi:10.3390/cells9071589)
Supplement: Supplementary file 1 [file cells-09-01589-s001.pdf]

cells MDPI

Oxidative inactivation of the proteasome augments alveolar macrophage secretion of vesicular SOCS3

Mikel D. Haggadone, Peter Mancuso and Marc Peters-Golden

Materials included: Supplementary materials and methods and supplementary figures S1-S9

Supplementary materials and methods

LDH assay

Toxicity of primary AMs and MH-S cells was calculated using CyQUANT LDH Cytotoxcity Assay (Thermo Fisher Scientific). After centrifugation of CM to remove dead cells, cell debris, and apoptotic bodies, 50 μL aliquots were collected to measure extracellular LDH release. To determine maximal measurable cytotoxicity, lysis buffer was added to cells for 20 h in parallel to the protocol described above for treatment of cells with CSE, H_2O_2, or bortezomib.

Quantification of MVs by flow cytometry

Flow cytometry was performed using a BD Biosciences (Franklin Lakes, NJ, USA) LSRFortessa. After centrifugation of CM as described above to remove dead cells, cell debris, and apoptotic bodies, 150 μL aliquots were incubated with 5 μL annexin V-FITC (BioLegend, San Diego, CA, USA) for 20 min in the dark at room temperature. Using 1 μm beads of known concentration (Thermo Fisher Scientific), MVs were determined as annexin V^+ particles having a diameter < 1 μm, and MV concentration in CM was calculated by determining the fraction of beads analyzed. Data were generated using FlowJo software (Version 10.5.0, BD Biosciences). To measure vesicular SOCS3 packaging by flow cytometry (vesicular SOCS3/MVs), the densitometric value for > 100 kDa SOCS3 (relative to control) was divided by the number of MVs quantified (% of control) in an aliquot collected from the same sample prior to 100-kDa centrifugal filtration.

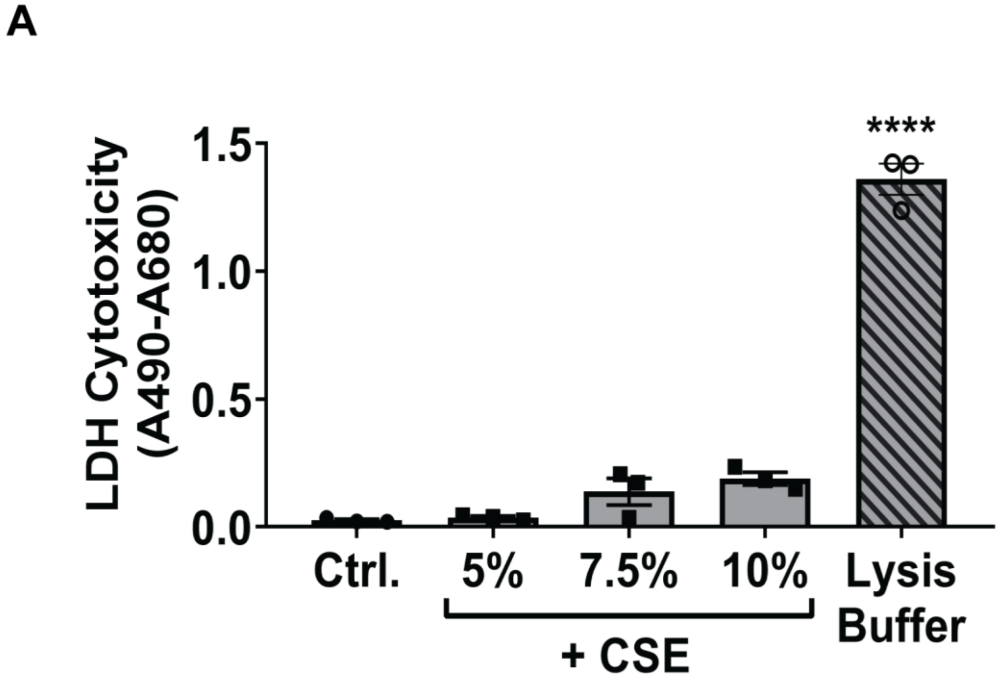

Figure 1. CSE does not cause toxicity in primary AMs. *A*, Adherent AMs collected by lung lavage were treated with specified concentrations of CSE for 1 h. Following CSE stimulation, AMs were washed and incubated for 20 h. CM aliquots were obtained for measurement of extracellular LDH, and maximal measurable LDH release was determined by incubating AMs with lysis buffer for 20 h. Data (mean ± SEM) are representative of 3 independent experiments with each condition analyzed in technical duplicates. Significance was determined by one-way ANOVA. Ctrl. = control. ****, $p < 0.0001$ *versus* control. "Circles" indicate data points for Ctrl. samples, "squares" indicate data points for CSE samples, and "open circles" indicate data points for lysis buffer samples.

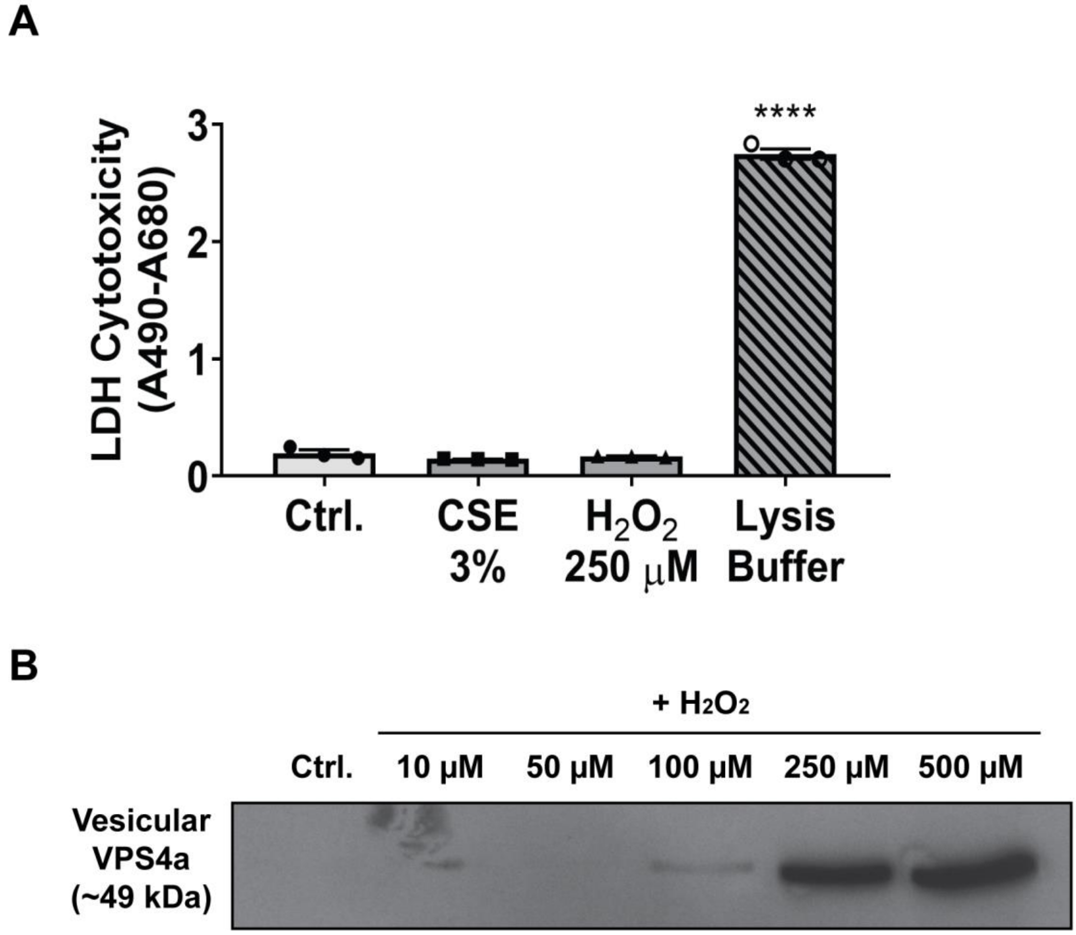

Figure 2. ROS do not cause toxicity in MH-S cells but promote release of vesicular VPS4a. (**A**) Adherent MH-S cells were treated with CSE (3%) or H_2O_2 (250 μM) for 1 h. Following treatment, cells were washed and incubated for 20 h. CM aliquots were obtained for measurement of extracellular LDH, and maximal measurable LDH release was determined by incubating cells with lysis buffer for 20 h. Data (mean ± SEM) are representative of 3 independent experiments with each condition analyzed in technical duplicates. Significance was determined by one-way ANOVA. (**B**) Adherent MH-S cells were treated with specified concentrations of H_2O_2 for 1 h. Following treatment, cells were washed and cultured for 20 h. Vesicular fraction samples were harvested and probed for VPS4a by western blot. Data are from 1 experiment representative of 3 independent experiments. Ctrl. = control. ****, $p < 0.0001$ *versus* control. "Circles" indicate data points for Ctrl. samples, "squares" indicate data points for CSE samples, "triangles" indicate data points for H_2O_2 samples, and "open circles" indicate data points for lysis buffer samples.

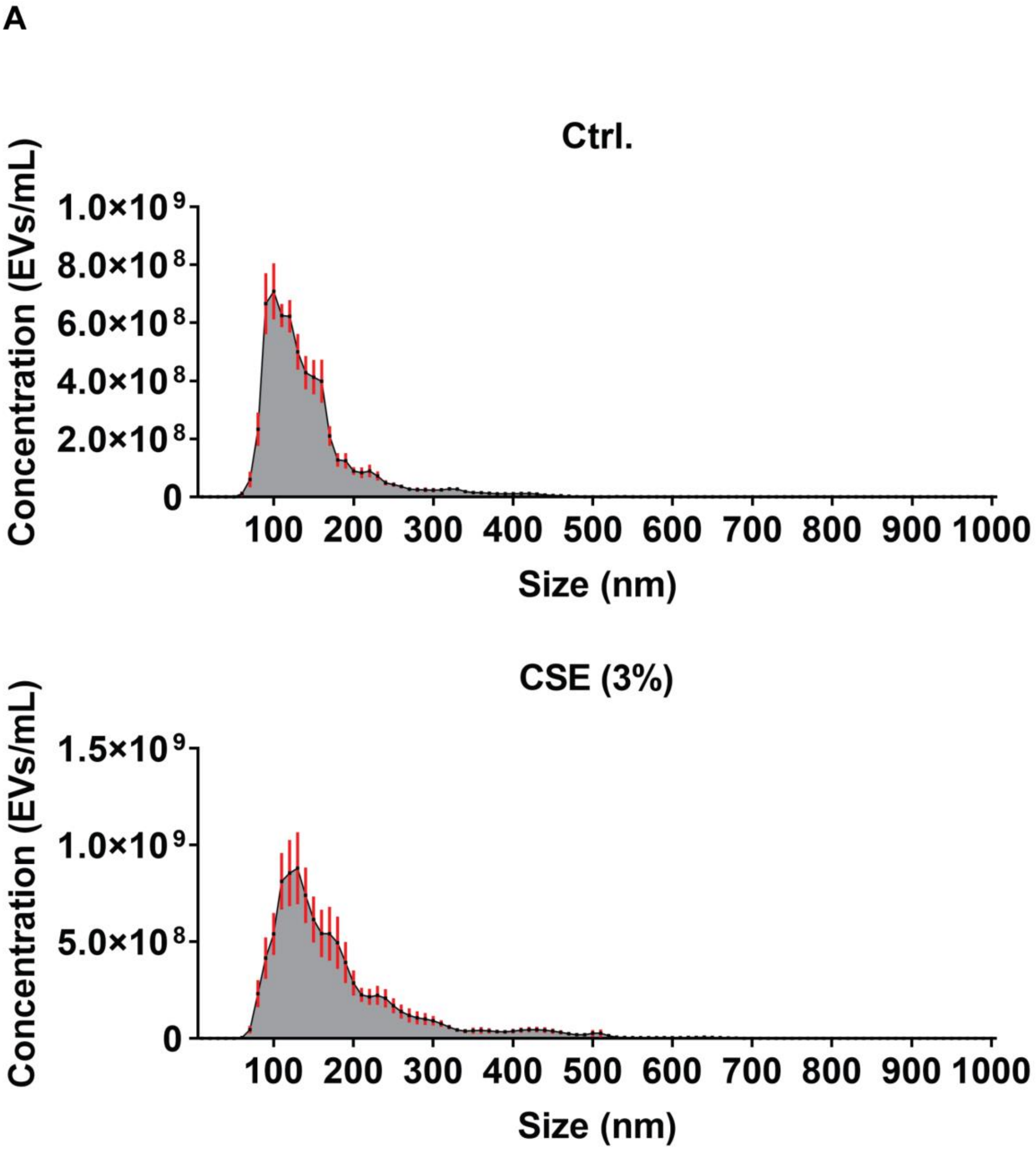

Figure 3. Histograms of NTA data collected for MH-S cells stimulated with CSE. *A*, Adherent MH-S cells were treated (1 h) with CSE, washed, and cultured for 20 h. EVs were collected by 100-kDa centrifugal filtration, diluted in PBS, and quantified by NTA. Data (mean ± SEM) are from 3 independent experiments in which ≥ 3 capture periods (60 s) were analyzed for each sample. Ctrl. = control.

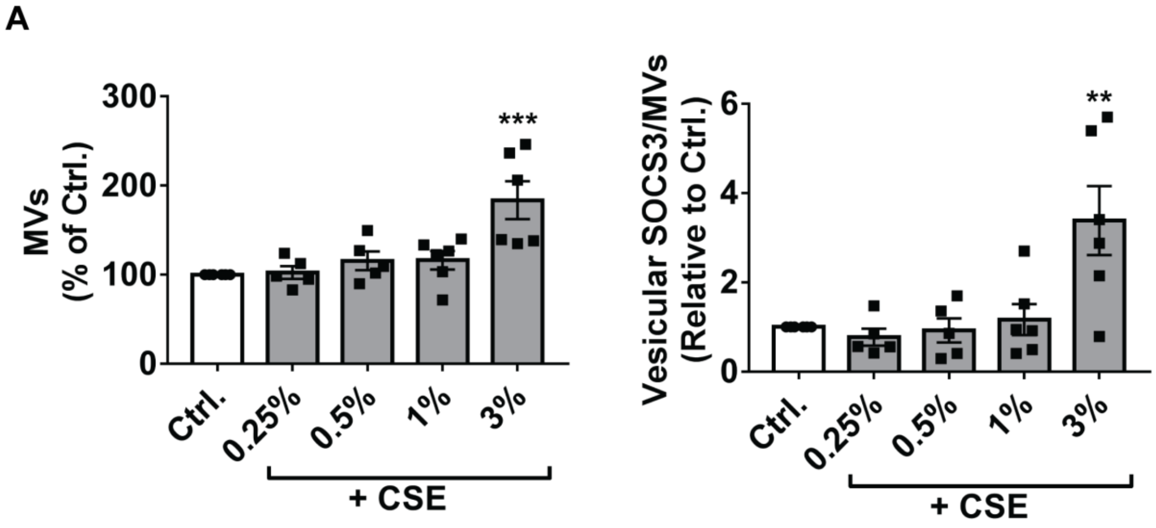

Figure 4. Flow cytometric analysis of CSE effects on MV production and vesicular SOCS3 packaging by MH-S cells. *A*, Adherent MH-S cells were treated for 1 h with indicated concentrations of CSE, washed, and cultured for 20 h. Aliquots were obtained from CM for quantification of MVs by flow cytometry (*left panels*). EVs were then concentrated in remaining CM samples by 100-kDa centrifugal filtration for probing of secreted vesicular (> 100 kDa) SOCS3. SOCS3 packaging was then determined, as described in "Experimental procedures" (*right panel*). Data (mean ± SEM) are from ≥ 3 independent experiments, and significance analyzed by one-way ANOVA. Ctrl. = control. ** and ***, $p < 0.01$ and $p < 0.001$, respectively *versus* control. "Circles" indicate data points for Ctrl. samples and "squares" indicate data points for CSE samples.

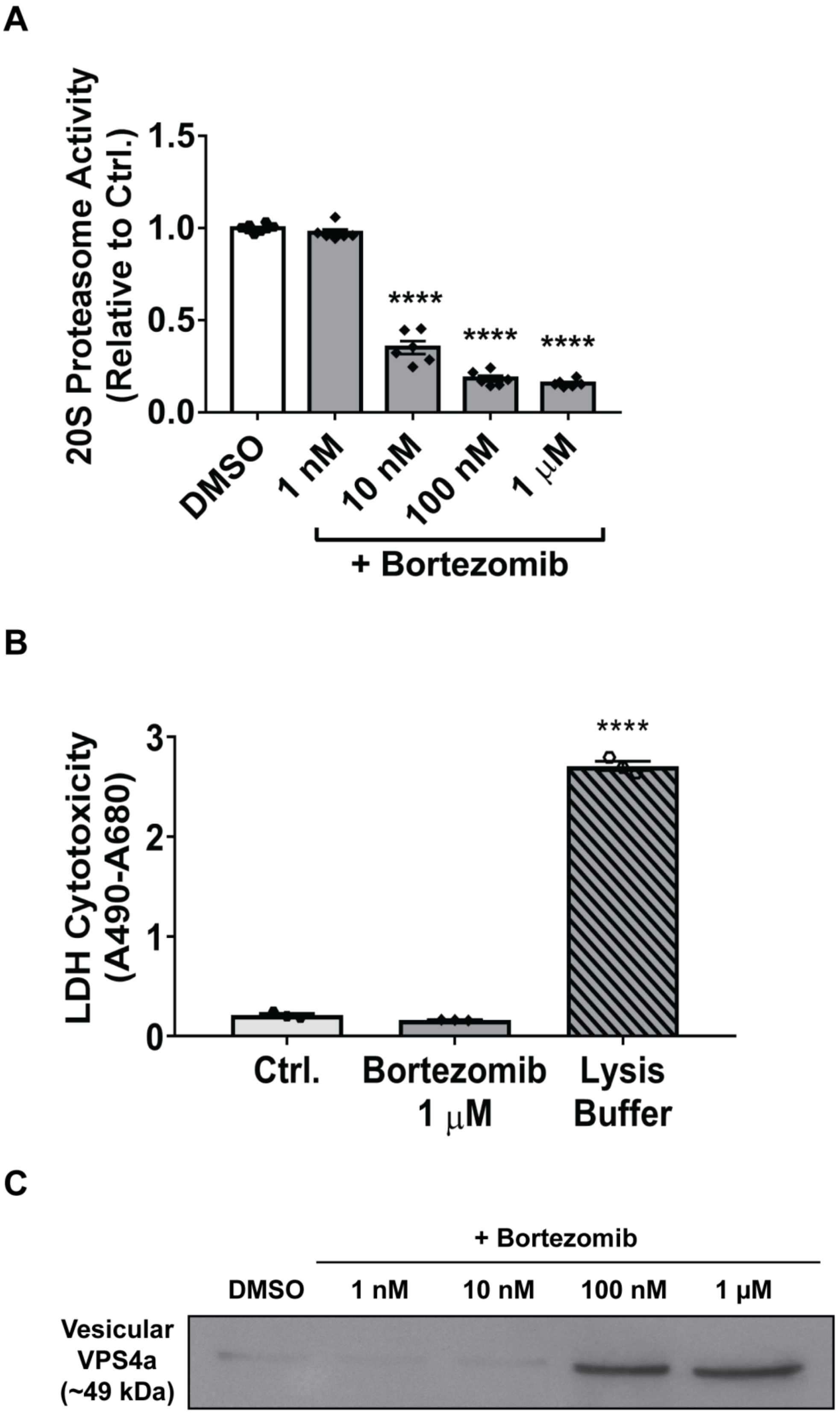

Figure 5. Bortezomib dose-dependently inhibits the proteasome and augments release of vesicular VPS4a without causing toxicity in MH-S cells. (**A-B**) Adherent MH-S cells were treated with the specified concentrations of bortezomib for 20 h. (**A**) Lysates were collected and proteasome activity was determined. Duplicate samples were analyzed for each condition, and data (mean ± SEM) are from 3 independent experiments. Significance was determined by one-way ANOVA. (**B**) CM aliquots were obtained for measurement of extracellular LDH, and maximal measurable LDH release was

determined by incubating cells with lysis buffer for 20 h. Data (mean ± SEM) are representative of 3 independent experiments with each condition analyzed in technical duplicates, and significance determined by one-way ANOVA. (**C**) Adherent MH-S cells were treated with specified concentrations of bortezomib for 20 h. Vesicular fraction samples were harvested and probed for VPS4a by western blot. Data are from 1 experiment representative of 2 independent experiments. DMSO = DMSO control. ****, $p < 0.0001$ *versus* DMSO control. "Hexagons" indicate data points for DMSO samples, "diamonds" indicate data points for bortezomib samples, and "open circles" indicate data points for lysis buffer samples.

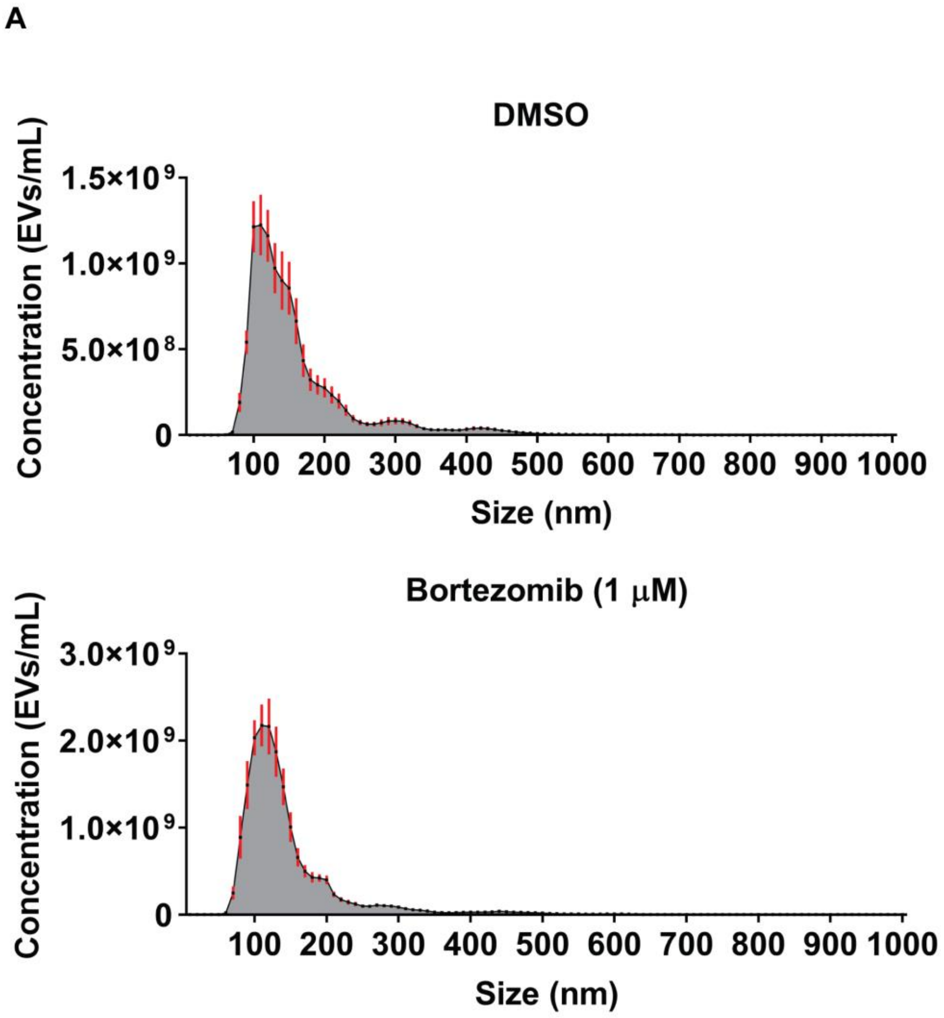

Figure 6. Histograms of NTA data collected for MH-S cells treated with bortezomib. *A*, Adherent MH-S cells were treated (20 h) with bortezomib (1 μM). EVs were collected by 100-kDa centrifugal filtration, diluted in PBS, and quantified by NTA. Data (mean ± SEM) are from > 3 independent experiments in which ≥ 3 capture periods (60 s) were analyzed for each sample. DMSO = DMSO control.

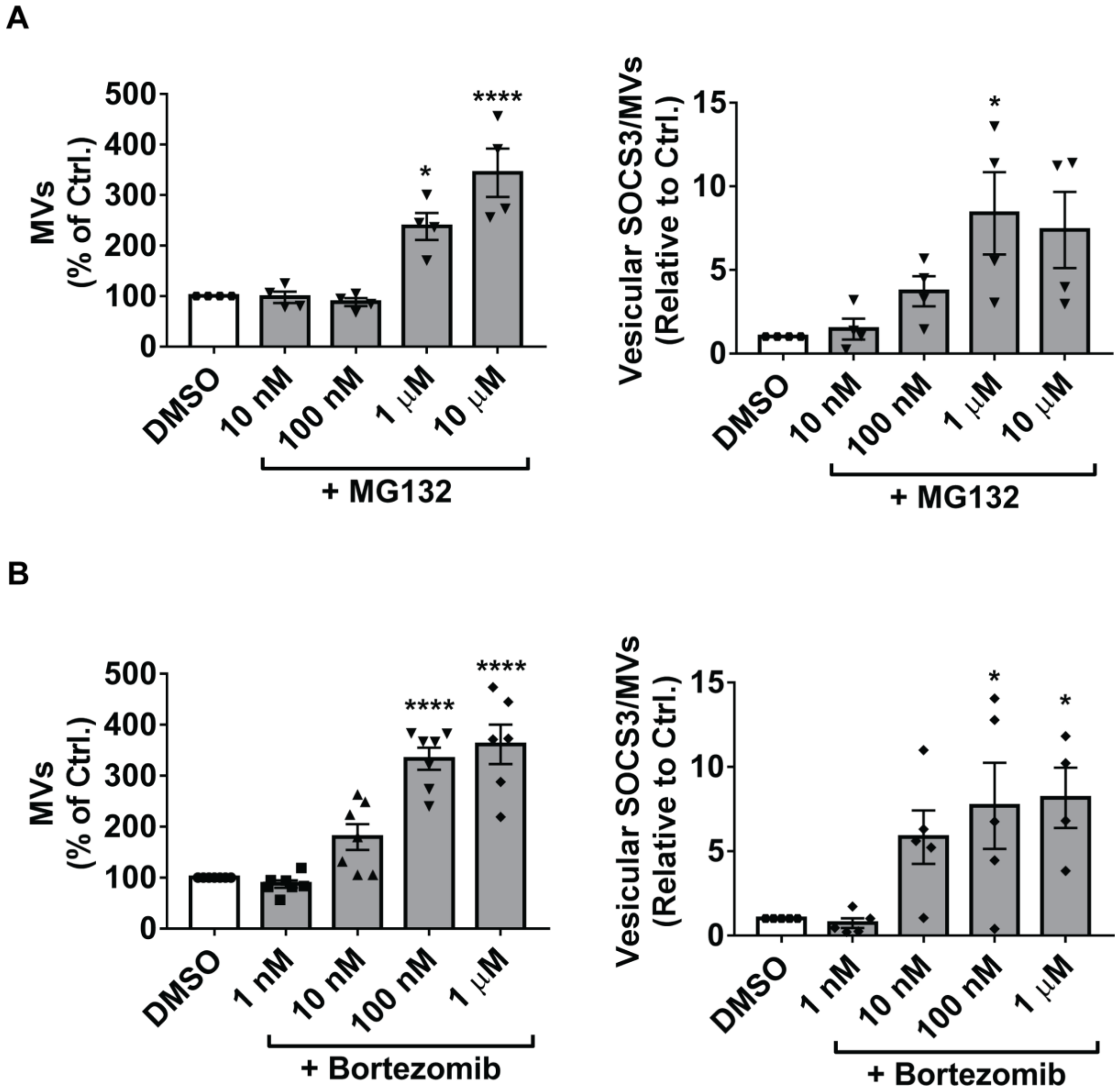

Figure 7. Flow cytometric analysis of proteasome inhibitor effects on MV production and vesicular SOCS3 packaging by MH-S cells. (**A-B**) Adherent MH-S cells were treated for 20 h with indicated concentrations of MG132 or bortezomib. Aliquots were obtained from CM for quantification of MVs by flow cytometry (*left panels*). EVs were then concentrated in remaining CM samples by 100-kDa centrifugal filtration for probing of secreted vesicular (> 100 kDa) SOCS3. SOCS3 packaging was then measured, as described in "Experimental procedures" (*right panel*). Data (mean ± SEM) are from ≥ 3 independent experiments, and significance analyzed by one-way ANOVA. DMSO = DMSO control. * and ****, $p < 0.05$ and $p < 0.0001$, respectively *versus* DMSO control. "Hexagons" indicate data points for DMSO samples, "inverted triangles" indicate data points for MG132 samples, and "diamonds" indicate data points for bortezomib samples.

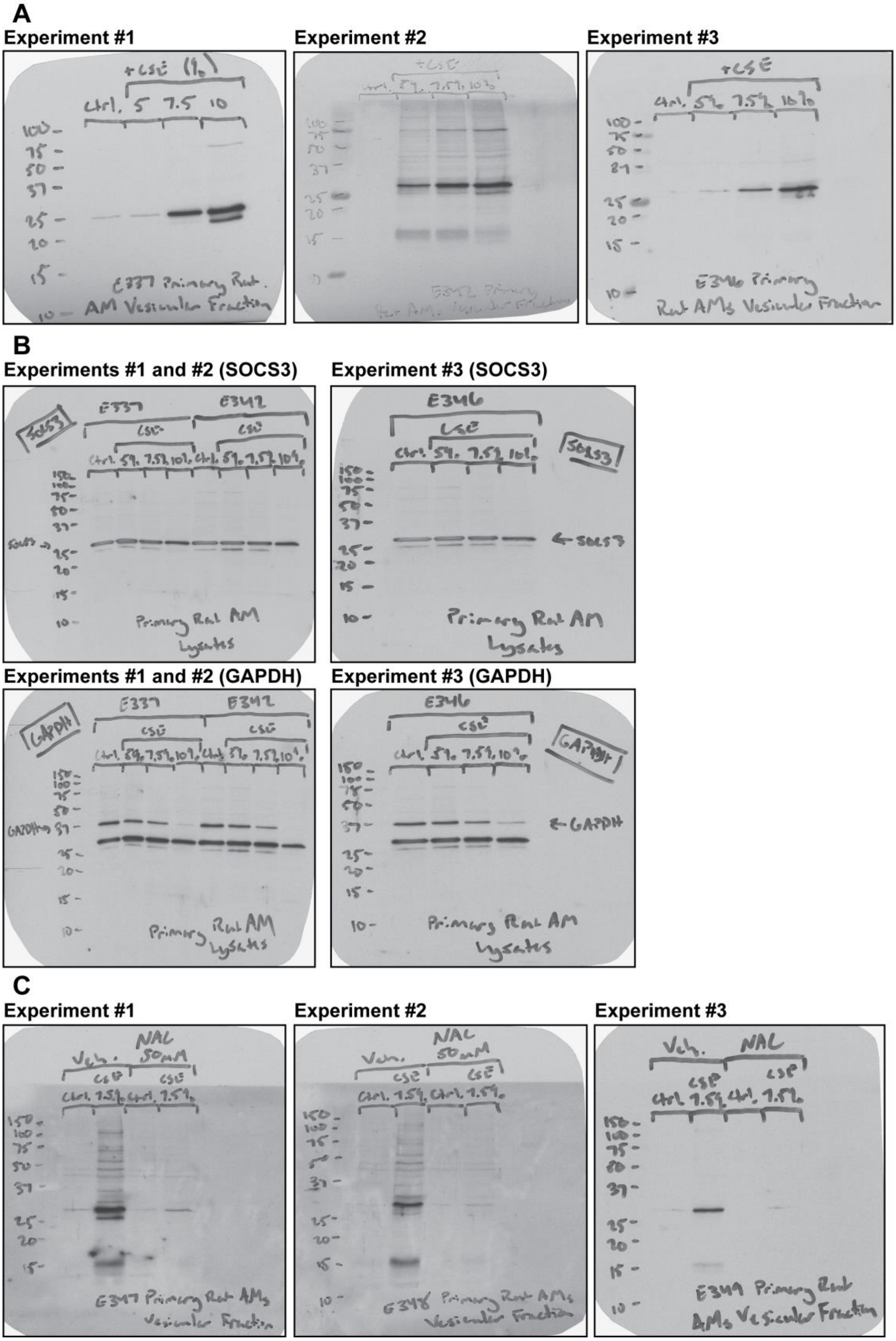

Figure 8. Original western blot images for primary AM data comprising Figure 1. (**A**) Replicate experiments for vesicular SOCS3 western blot data included in Figure 1A. (**B**) Replicate experiments for lysate SOCS3 and GAPDH western blot data included in Figure 1B. Note that membranes used to probe for GAPDH (37 kDa) were not stripped after probing for SOCS3 (27 kDa) resulting in the presence of two distinct bands. (**C**) Replicate experiments for vesicular SOCS3 western blot data included in Figure 1C.

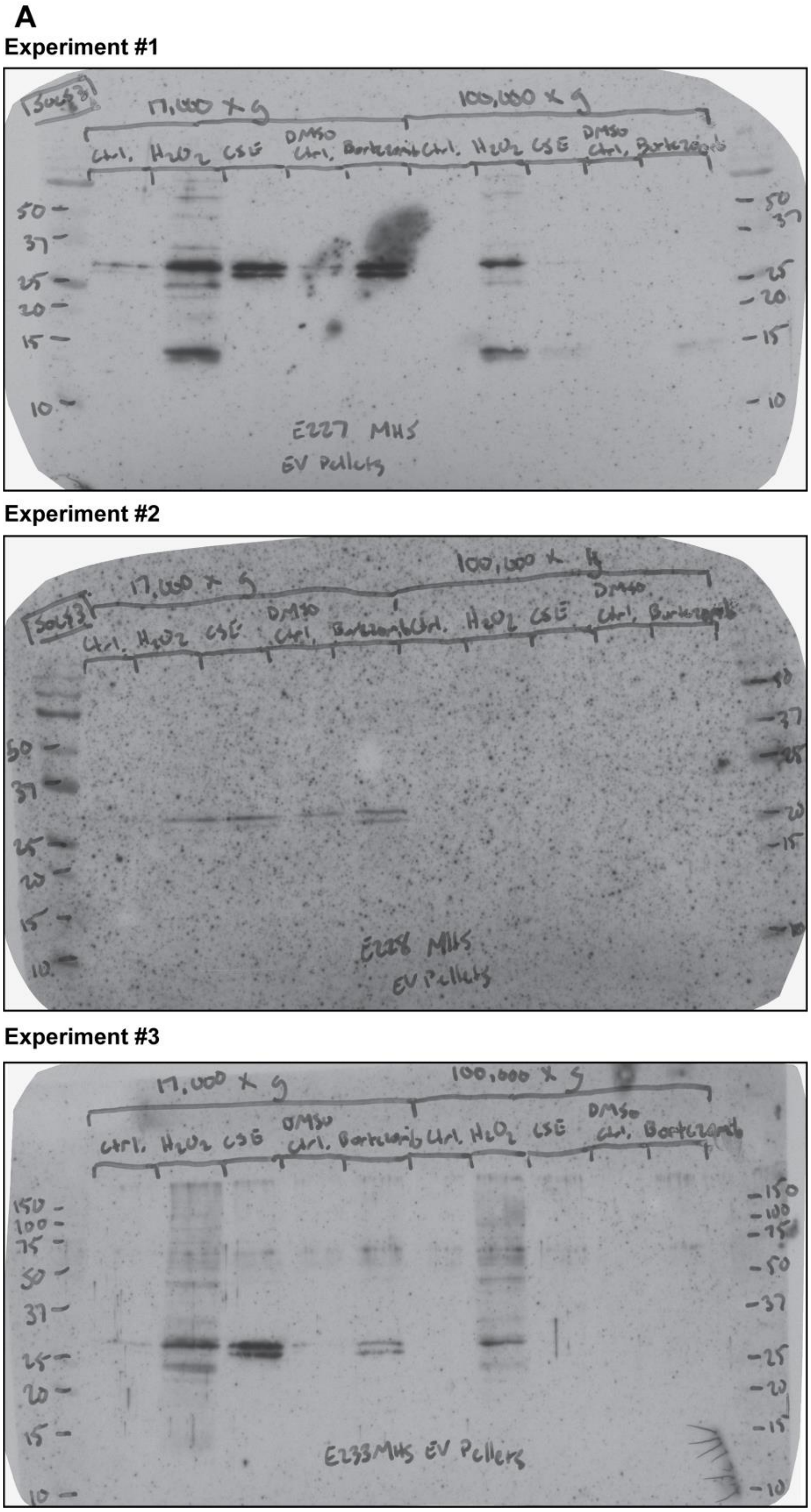

Figure 9. Original western blot images for MH-S cell data comprising Figures 3C and 6C. *A*, Replicate experiments for 17,000× *g* and 100,000× *g* SOCS3 western blot data included in Figures 3C and 6C. Note that H_2O_2 treatment data (lanes 2 and 7) were spliced from results included in Figure 3C.
